# Students’ Perception of Formative Assessment as an Instructional Tool in Competency-Based Medical Education: Proposal for a Proof-of-Concept Study

**DOI:** 10.2196/41626

**Published:** 2023-03-20

**Authors:** Farah Otaki, Mandana Gholami, Iman Fawad, Anjum Akbar, Yajnavalka Banerjee

**Affiliations:** 1 College of Medicine Mohammed Bin Rashid University of Medicine and Health Sciences Dubai United Arab Emirates; 2 Strategy and Institutional Excellence Mohammed Bin Rashid University of Medicine and Health Sciences Dubai United Arab Emirates; 3 Centre of Medical Education University of Dundee Dundee United Kingdom

**Keywords:** medical education, formative assessment, summative assessment, student, education, competency-based, proof-of-concept, perception, biochemistry, curriculum, teacher, educator, medical school, skill assessment, knowledge assessment, knowledge evaluation

## Abstract

**Background:**

In competency-based medical education (CBME), “Assessment for learning” or “Formative Assessment” (FA) plays a key role in augmenting student learning. FAs help students to measure their progress over time, enabling them to proactively improve their performance in summative assessments. FAs also encourage students to learn in a way where they address their knowledge gaps and gaps in their conceptualization of the subject matter. The effectiveness of an FA, as a learning and development instrument, relies on the degree of student involvement in the corresponding educational intervention’s design and implementation. The extent of students’ engagement in FA can be evaluated by appraising their perception regarding the educational intervention itself.

**Objective:**

This proof-of-concept study aims to develop a systemic understanding of a Formative Assessment as an Instructional Tool (FAIS) implemented in a biochemistry course in the Basic Medical Sciences component of an undergraduate entry, CBME.

**Methods:**

The educational intervention in question is an FAIS, which is implemented in a biochemistry course in the first semester of a 6-year bachelor of medicine, bachelor of surgery program. When developing the FAIS, each area of knowledge, skills, and attitudes were considered. Assessment formats are developed per Miller’s learning pyramid. This multiphase study is meant to rely on a convergent mixed methods design, where qualitative and quantitative data are independently collected and analyzed. Thereafter, the outputs of analyses are systematically merged using joint display analysis process. Qualitative data are collected through a focus group session that captures the students’ perception toward the FAIS. Data collection, integral to this focus group session, is exploratory. The inductive qualitative data analysis follows Braun and Clarke’s 6-step framework. The quantitative component of this study revolves around investigating the effect of the FAIS on the course’s summative assessment. The summative assessment performance of the 71 students, enrolled in the FAIS cohort, will be compared to that of the students in the non-FAIS cohort. The total duration of the proposed multiphase research study is 6 months.

**Results:**

This proposed multiphase study is expected to showcase, from a systemic perspective, the effectiveness of the respective educational intervention. It will shed light on the participating students’ attitudes in relation to the usefulness of FA in achieving competency goals and in fostering self-directed learning. The proposed study could also uncover the hypothesized association between the FA intervention and enhanced performance in summative assessments.

**Conclusions:**

Our findings will generate evidence regarding the application of FAs, which can be leveraged by other medical educators in contexts similar to those under investigation.

**International Registered Report Identifier (IRRID):**

DERR1-10.2196/41626

## Introduction

### Background

A pivotal challenge in implementing competency-based medical education (CBME) is the consequential assessment of competence. The transfer to CBME has raised awareness of the confines and limitations of existing assessment methods [1]. It has also highlighted the need to develop approaches to assess the competencies expected of today’s physicians in an era portrayed by increasing interdependence and collaboration among health care professionals, the recognition that patients’ safety is everyone’s responsibility, and an expectation of pellucidity and accountability.

In designing an assessment program, the intention is to articulate its purpose. Assessment has been shown to influence students’ excellence and allocation of their efforts. While experts might differ with regard to when and how the effects of assessment are exerted, the existence of such a relationship is irrefutable [2]. The influence of assessments on students’ learning, often referred to as the “educational impact” of the assessment, “testing effect,” “consequential validity,” “test-enhanced learning,” “backwash,” “washback,” and “testing phenomenon,” is an imperative element of the effectiveness of an assessment system [3]. Two fundamental and essentially different rationales are “assessment of learning” and “assessment for learning.” “Assessment of learning” or “Summative Assessment” evaluates a student’s learning progress and archetypally provides concrete grades or other objective measures [4]. The intended purpose of summative assessments is to measure the students’ achievement to make decisions about promotion or progression, direct what and how students learn, and motivate students to learn [5].

“Assessment for learning” or “Formative Assessment” (FA) is used to bolster the learning process. It focuses on providing the student with feedback on their performance to improve their skills [6], knowledge, and learning behavior. FAs focus on specific content, topics, and skills, and can be conducted as frequently as needed.

**Figure 1:**
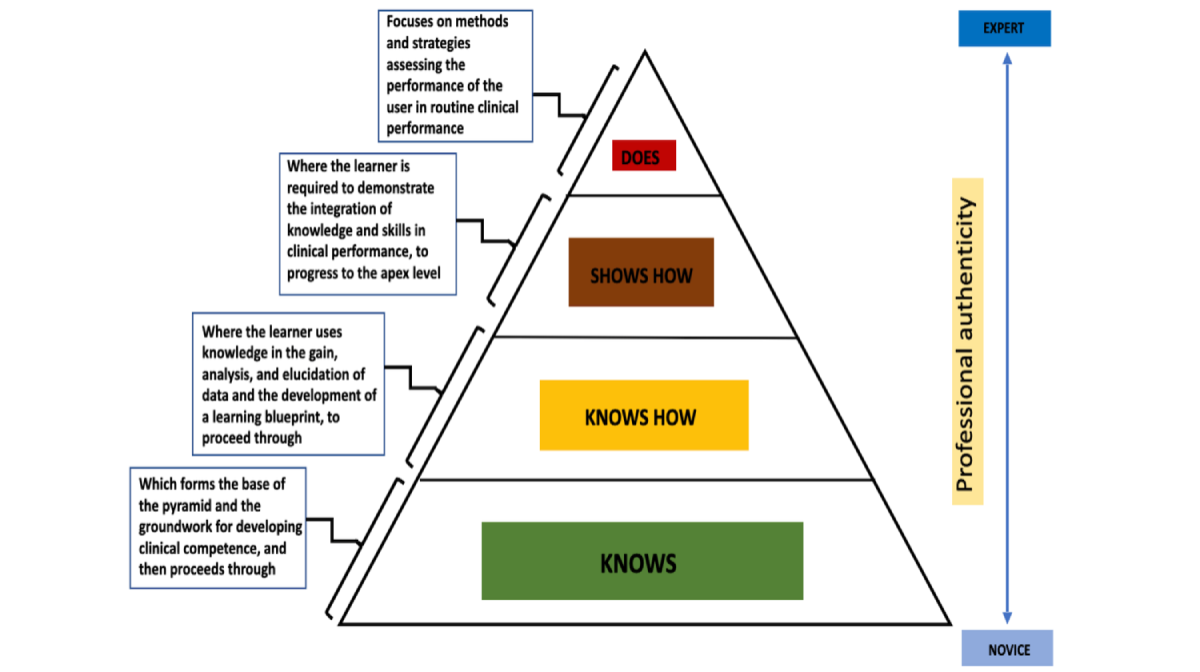
Miller's pyramid of learning [7].

FAs also serve the purpose of measuring students’ progress over time, which can assist the students to incessantly improve and support students in focusing their learning efforts on gaps in knowledge and their conceptualization of the subject matter. The key objectives of FAs are to inform the students of required knowledge for the future, identify student’s lag in performance, assist with timely performance improvement, and provide feedback to teachers about how best to assist and facilitate student learning. Miller [7] identified 4 levels of learning, conceptualized as a pyramid as shown in Figure 1. Beginning at the base, the learner “knows,” which forms the base of the pyramid and the groundwork for developing clinical competence, and then proceeds through “knows how,” where the learner uses knowledge in the gain, analysis, and elucidation of data and the development of a learning blueprint, to proceed through “shows how,” where the learner is required to demonstrate the integration of knowledge and skills in clinical performance, to progress to the apex level “does,” which focuses on methods and strategies to assess the performance of the user in routine clinical performance.

The assessment strategies tied to each level inform and contribute to learning as well as assessment, provided that formative feedback is given. At the “does” level, assessment becomes part of the authentic context in which one works and learns; learning provides deeper meaning for the trainee and builds a substrate for the cognitive processes of clinical decision-making [8].

Key to the effectiveness of an FA as a learning and development instrument is the degree of student involvement in the design and implementation of the FA used to train them. The extent of students’ engagement in FA can be effectively evaluated by appraising the students’ perception regarding the educational intervention itself.

Five specific features are essential to secure a sufficient level of student involvement: (1) congruence (ie, the tasks of an FA must reflect the instructional content), (2) authenticity (ie, the tasks of an FA should be related to students’ backgrounds and study contexts), (3) consultation (ie, students should have a say in how their answers are evaluated and based on which criteria), (4) transparency (ie, no “mystery items”; the wording of the items must clearly address the targeted content), and (5) accommodation (ie, all students should have the same chance to complete the items of a FA).

### Objective of the Proposed Study

The overall objective of the multiphase proof-of-concept study proposed in this protocol is to develop a systemic understanding of the effectiveness of a Formative Assessment as an Instructional Tool (FAIS) implemented in a biochemistry course in the Basic Medical Sciences component of an undergraduate entry, competency-based medical curriculum at the Mohammed Bin Rashid University (MBRU) of Medicine and Health Sciences, Dubai, the United Arab Emirates.

## Methods

### Study Context

The CBME at MBRU comprises 3 phases. Each phase of the curriculum includes integrated courses and builds on the preceding one, such that the curriculum is “spiral.” The students purposefully repeat subjects, where with each successive encounter, concepts build upon those acquired in the previous round. The medical school, in which the proposed multiphase study will be conducted, caters to a student population from more than 19 different countries and from 20 different high school curricula. Approximately 75% of the students are female [9].

### Research Team

CBME requires medical educators to reimagine almost every aspect of undergraduate medical training, including curriculum delivery and assessments. Therefore, the proposed multiphase study is meant to be conducted through a reliable student-faculty partnership, which is expected to facilitate the entailed processes. This includes involving students as coinvestigators, where they are considered partners in the entailed decision-making processes, through their active engagement in collaborative meetings. The 3 student researchers who are involved in the proposed multiphase study include MG, IF, and AA, who are drawn from across the spectrum of the respective medical curriculum. Further, as the proposed study forms a part of a process whereby MBRU engages in continuing self-evaluation to measure achievements and outcomes as they relate to the institution’s preset goals (ie, effectiveness of MBRU as an institution in disseminating medical and health sciences education), a member of the strategy and institutional excellence (SIE) team (ie, the entity handling the MBRU Quality Assurance and Institutional Effectiveness portfolio), FO, is part of the team as a co–principal investigator. She is a senior specialist in the SIE unit with expertise in research design and quantitative and qualitative data analyses. Her research interest is focused on means of nurturing health professionals to equip them with competences complementary to basic and clinical medical sciences and how this affects the health professionals’ clinical performance and, in turn, outcomes of care. The global aspect of this study is overseen by YB. His research expertise in medical education is primarily focused on epistemology, ethnography, delineation of strategies for integration, and contextualization of basic sciences in the medical curriculum to inform clinical practice, change-management models, and Pierre Bourdieu’s multifaceted concept of habitus with the aim to understand and transform how groups with diverse clinical proficiency work together to improve patient outcomes. The involvement of a multidisciplinary student-partnered team is, therefore, meant to raise the reliability of the proposed research project and its findings.

### The Educational Intervention Under Investigation

The FAIS was implemented in a biochemistry course in the first semester of a 6-year bachelor of medicine, bachelor of surgery program. The reason the FAIS was implemented in this course is because one of the key aims of this course is to develop explicit associations among basic research, medical understanding, and the patients’ perspectives. When developing the FAIS, each area of knowledge, skills, and attitudes was considered. Assessment formats were developed in alignment with the Miller’s learning pyramid (Figure 1) [7]. The “student knows” and “student knows how” were assessed with multiple-choice questions and long cases. “Student shows” was assessed with laboratory examinations and objective structured practical examinations. “Student does” assessed the students’ competences of analysis of biochemical data. The assessments used to assess “student knows,” “student knows how,” and “student shows” were standardized assessments. “Student does” level assessments were unstandardized.

### Research Design

The multiphase study, proposed in this protocol, is meant to rely on a convergent mixed methods design [10], where the qualitative and quantitative data are independently collected and analyzed. Following that, the output of analysis is systematically merged using joint display analysis process [11,12]. Integration of the output of analyses, as such, is expected to raise the reliability of the study findings, enabling the generation of a holistic understanding of the subject matter. The study comprises 4 phases: data collection, data analyses, information integration, and knowledge generation (Table 1).

**Table 1:** Outline of the design of the proposed proof-of-concept study.

Phase 1: data collection	Phase 2: data analyses	Phase 3: information integration	Phase 4: knowledge generation
Qualitative: focus group session	Braun and Clarke’s [13] 6-step framework	Joint display analysis to generate meta-inferences	Interpretation of study findings
Quantitative: students’ performance in the summative assessment	ANOVA with a *P* value of ≤.05	Joint display analysis to generate meta-inferences	Interpretation of study findings

### Ethical Considerations

Since the proposed study entails “no more than minimal risk” on human participants, and all the data are already collected as part of institutional research functions and handled by the SIE team at MBRU (which adhere, by virtue of design, to the ethical principles of autonomy, justice, and beneficence), an exempt review will suffice. Progressing with the analyses, reflected upon in this protocol (namely qualitative and quantitative analyses and joint display analysis), is contingent upon the exempt review clearance of the institutional review board of MBRU.

### Evaluation of Students’ Perceptions

The qualitative component of the study, as illustrated in Table 1, revolves around carrying out a focus group session [14-16] composed of 7-10 randomly selected students from the cohort of 71 students who were exposed to the FAIS. One of the study investigators, who is experienced in socio-behavioral research and was not involved in planning or implementing the FAIS, facilitated the focus group session based on a preset focus group protocol designed for this study (Multimedia Appendix 1). Prior to conducting this focus group session, the protocol underwent face and content validation. The students’ participation in this focus group session was completely voluntary, and each participant was required to provide verbal consent prior to the commencement of the session. The data collection, integral to this component of the study, was exploratory, where the participants were invited to externalize their thoughts in relation to their individualized experiences with the FAIS.

Student investigators attended the focus group session in order to ensure flow of the entailed discussions and to take notes and arrange for the recording of the session. The inductive qualitative data analysis will follow Braun and Clarke’s [13] 6-step framework recently endorsed in health professionals’ education research [17].

### Evaluation of the Impact of the FAIS on Students’ Performance in Summative Assessments

The quantitative component of the study, as illustrated in Table 1, revolves around the investigation of the effect of the FAIS on the students’ performance in the summative assessment. As such, the performance in the summative assessment of the 71 students enrolled in the cohort that was exposed to the FAIS will be compared to that of the students enrolled in a cohort that was not exposed to the FAIS. ANOVA will be performed with the scores of the summative assessments of all students enrolled in both cohorts. A *P* value of ≤.05 will be considered the level of significance.

### Joint Display Analysis

The output of analyses of the qualitative and quantitative components of this study (ie, first level of inferences), as illustrated in Table 1, will be integrated using the iterative joint display analysis process [11,12], which enables the development of a macrolevel understanding of the subject matter.

### Study Plan

The total duration of the proposed study is 6 months. The key milestones and time line are shown in Table 2.

**Table 2:** Plan of the proposed multiphase study.

Milestones	Month 1	Month 2	Month 3	Month 4	Month 5	Month 6
Planning for the formative assessment intervention	✓^a^					
Developing and validating the focus group protocol	✓					
Implementing the formative assessment intervention		✓	✓	✓		
Recruiting study Participants				✓		
Conducting the focus group session					✓	
Analyzing data (qualitative and quantitative, and joint display analysis process)					✓	
Reporting on generated information					✓	
Preparing for knowledge sharing						✓

^a^✓: indicates completion of the task at the set deadline.

## Results

The mixed methods study proposed in this protocol is expected to generate a systemic understanding of the effectiveness of the respective FAIS educational intervention. This involves exploring the students’ perceptions regarding the FAIS, along with investigating the intervention’s effectiveness in enhancing the participating students’ performance in the corresponding summative assessment. It will shed light on the participating students’ attitudes in relation to the use of FA in achieving competency goals and in fostering self-directed learning. It could also uncover the hypothesized association between the FA intervention and enhanced performance in summative assessments.

## Discussion

### Anticipated Findings

A systemic understanding of the effectiveness of the FAIS in enhancing academic performance and enabling self-directed learning will be developed through the proof-of-concept study proposed in this protocol. The multistage inductive qualitative analysis is expected to generate a conceptual framework that describes the educational intervention from the viewpoint of the learners. The respective framework is expected to highlight the students’ reactions and the perceived effects of the FAIS (short- and long-term) and opportunities for improvement. The quantitative component is meant to investigate the association between the FAIS and academic performance (in the summative assessment). The joint display analysis will reveal the lessons learned from the firsthand implementation of the FAIS in the context of the study, and the factors (ie, enablers) that need to be taken into account to maximize the value of such an educational intervention to all involved parties.

Within the context of the study, our findings will shed light on opportunities to maximize the value of the educational intervention, especially in relation to engaging and empowering the students and fostering self-directed learning. The perception of the students of the experience will be factored into the design of upcoming rounds of the FAIS, which is expected to raise the reliability of such interventions [18]. Additionally, if the outcomes are favorable, the long-term goal is to implement the designed and improved FA intervention across all courses in the program under investigation, for which we will strategize a change management approach using Mento’s change management model [19].

Self-directed learning has gained widespread recognition [20]. Medical educators have been experimenting with differing techniques to foster self-directed learning and in turn lifelong learning habits [18,21,22]. The study proposed in this protocol is likely to prove that the FAIS is effective in enhancing academic performance through fostering self-directed learning [23,24]. It is expected to enable learners to develop self-regulatory strategies by promoting cognition (ie, learning) and meta-cognition (ie, learning to learn) [25]. Hence, critical and higher-order thinking, along with integration of disciplinary knowledge, are likely to be improved [26]. This will enhance the students’ preparedness for summative assessment. It will be interesting to explore the students’ perceptions of the learning experience, since it is believed that active student engagement and learner agency can only be ensured when the learners (actually) perceive the benefits of the educational intervention. The proposed study is meant to suggest evidence-driven ways to make the true value of the educational intervention prominent to the learners.

### Limitations

The proposed study is characterized by certain limitations. The mixed methods research design, systematically integrating qualitative with quantitative data, enables the generation of thorough insights into the subject matter [27]. Yet, given that the FAIS is implemented in a single biochemistry course in the Basic Medical Sciences component of an undergraduate entry, competency-based medical curriculum at MBRU, the generalizability of the findings is limited. The context of the study is described in detail to enable the transferability of the findings to contexts similar to those under investigation. Also, the study design, especially how the quantitative component is structured, enables investigations of potential associations. Yet, it does not allow for the uncovering of causalities. It would be worthwhile for future studies to take longitudinal approaches that actually reveal the intricacies of the relationship of FAs and academic performance and other desired skills (eg, self-directed learning and academic resilience) [12], which may turn out to play a moderating (or even mediating) role.

### Conclusions

The findings of the proposed study constitute evidence in relation to the application of FAs, which can be leveraged by other medical educators in contexts similar to those under investigation.

## Appendix

Multimedia Appendix 1 Online Focus Group Protocol.

## Abbreviations

CBME: competency-based medical education
FA: formative assessment
FAIS: Formative Assessment as an Instructional Tool
MBRU: Mohammed Bin Rashid University
SIE: strategy and institutional excellence

## Data Availability

All data analyzed during this study are included in this published article (and its supplementary information files).
