# Supplementary material for: Students’ Perception of Formative Assessment as an Instructional Tool in Competency-Based Medical Education: Proposal for a Proof-of-Concept Study
Source: JMIR Res Protoc. 2023 Mar 20;12:e41626. doi: 10.2196/41626 (PMC10131604; doi:10.2196/41626)
Supplement: Multimedia Appendix 1 [file resprot_v12i1e41626_app1.pdf]

## Appendix I: Online Focus Group Protocol

Purpose: To explore students' perception regarding a Formative Assessment intervention implemented in a biochemistry course in the Basic Sciences component of an undergraduate medical program in Dubai, UAE (45 minutes).

### Part A: General understanding- Formative Assessment (5 minutes)

Structure, value, and feedback

General effect on:

Learning style (transition to/ from surface, deep, and/ or strategic)

Learning environment (including but not limited to relationship with tutor and peers)

### Part B: Impression/ perception of experience with this Formative Assessment (10 minutes)

Learning experience integral to this Formative Assessment (Assessment for Learning)

Description

Reaction: thoughts and emotions (be it negative and/ or positive)

Structure of this Formative Assessment

Selected format(s) (MCQ and short answers following a case), Extent of innovativeness, Frequency, and Allocated time

Feedback integral to this Formative Assessment

Format, Content, Timeliness, and Value

Sufficiency of guidance (how to address gap not only what/ where is the gap)

Proactiveness in this Formative Assessment

Academic Maturity [self-evaluation, and academic perceptiveness (knowing how best to learn and to leverage existing resources to one's own learning advantage) and motivation]

Self-directed learning, Willingness to maximize from experience, and Techniques adapted to maximize from experience

### Part C: Effects of this Formative Assessment (10 minutes)

Effect, of this Formative Assessment, on:

Learning style (transition to/ from surface, deep, and/ or strategic)

Learning environment (including but not limited to relationship with tutor and peers)

Value of this Formative Assessment

In relation to the six core competences of Medical Education

Immediate and expected long-term benefits

Effect of this Formative Assessment on performance

Student-instructor Relationship (e.g., communication and engagement)

### Part D: Opportunities to improve this Formative Assessment (5 minutes)
